# THE IMPLEMENTATION OF A STATEWIDE INTERDISCIPLINARY OLDER ADULT FINANCIAL JUSTICE COALITION

**DOI:** 10.1093/geroni/igae098.3146

**Published:** 2024-12-31

**Authors:** Briana Kohlbrenner, Jodi Catlow, Eric Chess, Carson De Fries

**Affiliations:** University of Denver, Denver, Colorado, United States; University of Denver, Denver, Colorado, United States; University of Denver, Denver, Colorado, United States; University of Denver, Denver, Colorado, United States

## Abstract

Financial fraud and exploitation have devastating impacts that are felt acutely among older adults. To address this issue in Colorado, a coalition was formed in March of 2023 to bring together the state’s strong but historically disconnected contingent of experts across disciplines. This coalition includes 60 individuals representing 30 agencies including law enforcement, financial institutions, adult protective services, government, and non-profit agencies. The goals of the coalition are to 1) convene and grow this network to include all key stakeholders and leaders in financial fraud and exploitation across the state, 2) provide support, training, actionable tools, and resources for front-line workers and 3) streamline resources and create a collaborative state-wide hub for resources around financial abuse. Through this initiative we have identified barriers to collaborative efforts in this area including sharing data across agencies, building trust between industries, and an overall lack of shared language. Lessons learned from the implementation of this coalition include how to communicate across disciplines, elevating the expertise of those in the field without replicating work or operating in silos, and utilizing an academic institution to facilitate these collaborative efforts. To provide an overview of the innovative and interdisciplinary coalition, we will showcase the process of developing, maintaining, and expanding this coalition with the purpose of providing a framework for other states to replicate.

